# Compliance to advanced trauma life support protocols in adult trauma patients in the acute setting

**DOI:** 10.1186/1749-7922-8-39

**Published:** 2013-10-02

**Authors:** Bonnie Tsang, Jessica McKee, Paul T Engels, Damian Paton-Gay, Sandy L Widder

**Affiliations:** 1 Department of Surgery, Faculty of Medicine and Dentistry, University of Alberta, 2D WMC, 8440-112 Street NW, Edmonton, AB T6G 2B7, Canada; 2 Alberta Centre for Injury Control and Research, School of Public Health, University of Alberta, Edmonton, AB, Canada; 3 Department of Surgery and Division of Critical Care, Faculty of Medicine and Dentistry, University of Alberta, Edmonton, AB, Canada

**Keywords:** Advanced trauma life support, Quality improvement, Trauma team leader, Major trauma, Wounds & injuries

## Abstract

**Introduction:**

Advanced Trauma Life Support (ATLS) protocols provide a common approach for trauma resuscitations. This was a quality review assessing compliance with ATLS protocols at a Level I trauma center; specifically whether the presence or absence of a trauma team leader (TTL) influenced adherence.

**Methods:**

This retrospective study was conducted on adult major trauma patients with acute injuries over a one-year period in a Level I Canadian trauma center. Data were collected from the Alberta Trauma Registry, and adherence to ATLS protocols was determined by chart review.

**Results:**

The study identified 508 patients with a mean Injury Severity Score of 24.5 (SD 10.7), mean age 39.7 (SD 17.6), 73.8% were male and 91.9% were involved in blunt trauma. The overall compliance rate was 81.8% for primary survey and 75% for secondary survey. The TTL group compared to non-TTL group was more likely to complete the primary survey (90.9% vs. 81.8%, p = 0.003), and the secondary survey (100% vs. 75%, p = 0.004). The TTL group was more likely than the non-TTL group to complete the following tasks: insertion of two large bore IVs (68.2% vs. 57.7%, p = 0.014), digital rectal exam (64.6% vs. 54.7%, p = 0.023), and head to toe exam (77% vs. 67.1%, p = 0.013). Mean times from emergency department arrival to diagnostic imaging were also significantly shorter in the TTL group compared to the non-TTL group, including times to pelvis xray (mean 68min vs. 107min, p = 0.007), CT chest (mean 133min vs. 172min, p = 0.005), and CT abdomen and pelvis (mean 136min vs. 173min, p = 0.013). Readmission rates were not significantly different between the TTL and non-TTL groups (3.5% vs. 4.5%, p = 0.642).

**Conclusions:**

While many studies have demonstrated the effectiveness of trauma systems on outcomes, few have explored the direct influence of the TTL on ATLS compliance. This study demonstrated that TTL involvement during resuscitations was associated with improved adherence to ATLS protocols, and increased efficiency (compared to non TTL involvement) to diagnostic imaging. Findings from this study will guide future quality improvement and education for early trauma management.

## Introduction

Trauma is the most common cause of death in Canada for the age group of 44 years or less. In 2004, intentional and unintentional injuries led to 13,677 deaths, and 211,000 hospitalizations [1]. The economic burden from injuries is estimated at $10.7 billion in health care costs, and $19.8 billion in total economic costs [1].

Trauma resuscitations often involve complex decision-making and management of critical injuries in a short span of time. Errors are common; an Australian study on trauma management found 6.09 errors per fatal case in the emergency department (ED) with 3.47 errors contributing to patient death [2]. Since 1977, the Advanced Trauma Life Support (ATLS) treatment paradigm was established to improve the management of trauma patients during the initial resuscitation phase [3]. ATLS protocols provide a common framework and organized approach during these situations, and have been shown to improve outcomes [4,5]. Unfortunately, attrition rate of ATLS knowledge [6,7] and low compliance rate are issues even in major trauma centers. Deviations from ATLS protocols are common, ranging from 23% to 53% [8-11]. Compliance rate can affect patient outcome [4,5], and can serve as a surrogate marker for quality assessment of a trauma system.

Adherence to ATLS protocols is only investigated by a few studies [9-11]; specifically, whether the presence or absence of a Trauma Team Leader (TTL) affects adherence. The primary objective of this study was to determine the compliance rate with ATLS protocols in the ED in a Canadian Level I trauma centre, as well as to assess the impact on ATLS compliance with TTL involvement. Secondary objectives included assessing patient outcomes and times to diagnostic imaging.

## Methods

This study was conducted in a Level I trauma center in Canada. Ethics approval for the study was obtained from the Human Research Ethics Review Board at the University of Alberta. Patients meeting inclusion criteria were identified from the Alberta Trauma Registry (ATR) from July 1, 2009 to June 30, 2010. Inclusion criteria were: age ≥17 years old, Injury Severity Score (ISS) ≥12, and patients with injuries that occurred <24 hours prior to presentation to the trauma centre. Patients with non-acute injuries (injuries sustained ≥24hrs), drowning, strangulations, missing charts and inter-hospital transfers that bypassed ED assessment were excluded.

The ATR collects data prospectively on all trauma patients with an ISS ≥12 who are admitted to one of the ten participating trauma centers in Alberta. Data obtained from the ATR included: date of injury, sex, age, mechanism of injury, discharge status, total length of stay (LOS), ICU (Intensive Care Unit) LOS, ISS, and revised trauma score (RTS). A retrospective chart review was performed for additional data not collected in the ATR, on the completion of various actions or tasks as per ATLS protocols (see Table 2), as well as time to diagnostic tests, readmission to hospital, and presence or absence of TTL during resuscitation. Readmission rate in this study included all unplanned readmissions to a hospital in Alberta within 60 days of discharge.

Criteria for trauma team and/or TTL activation

Respiratory distress

Hemodynamic instability

Focal neurological signs or GCS ≤8

Penetrating torso trauma

Multiple casualties

Major burn

At the discretion of the ED physician or charge nurse

At the time of the study, the core trauma team was composed of the TTL, senior and junior general surgery residents, orthopedic resident, anesthesia resident, along with nursing staff, radiology technicians, and respiratory therapists. Attending surgeons were available within 30 minutes while on-call. Other surgical specialties (neurosurgery, thoracics, vascular), intensivist, as well as hemoatologist were available upon request. The decision to activate the trauma team was based on criteria listed above. In cases where the trauma team was not activated, it was at the discretion of the ED physician in charge to consult the appropriate services.

TTLs were multidisciplinary and composed of emergency physicians, general surgeons, and one neurosurgeon. All of the TTLs have ATLS certification, and a strong interest in trauma. Members of the TTL group are involved in ATLS education, quality assurance, and research. For major traumas meeting criteria (see above), the TTL on-call was activated and was expected to arrive within 30 minutes to take over the leadership role of the resuscitation. When a TTL was not available, the leadership role fell onto the ER physician in charge, a senior surgical resident, or the general surgeon on call.

Two groups were created for the analysis: the TTL group and the non-TTL group. Basic demographic analysis was completed on the two groups involving age, sex, ISS, total LOS, ICU LOS, RTS, mechanism of injury and mortality. Chi square analysis was used to compare the ATLS protocol compliance between the two groups, as well as the mortality rate and readmission rate. Independent sample T-Test was used to compare the times to diagnostic imaging and Mann–Whitney U test (2 sample) was used to compare the number of items completed in the primary and secondary survey. Statistical analysis was performed using SPSS software, version 19 (IBM Corporation, Armonk, New York).

## Results

A total of 781 patients were identified from the ATR that met the inclusion criteria. Two hundred seventy three of the patients were excluded by criteria. A total of 508 patients were analyzed.

### Demographics

Of the 508 patients, mean age was 39.7 (SD 17.6), 375 (73.8%) were male, and the mean ISS was 24.5 (SD 10.7) (Table 1). The majority of the patients (n = 467, 91.9%) suffered blunt trauma, whereas penetrating trauma and burns accounted for 5.7% (n = 29) and 2.4% (n = 12) of the patients respectively. Overall mortality was 4.9% (n = 25).

**Table 1:** Patient demographics

	**All patients (n = 508)**	**TTL (n = 274)**	**Non-TTL (n = 234)**	**p-value**
Male	375 (73.8%)	210 (76.6%)	165 (70.5%)	0.117
Mean age (years)	39.7 (SD 17.6)	39.2 (SD 17.3)	40.3 (SD 18.0)	0.457
Mean ISS	24.5 (SD 10.7)	25.4 (SD 11.0)	23.5 (SD 10.2)	0.045
Mean ICU LOS (days)	3.7 (SD 9.0)	4.5(SD 9.8)	2.9 (SD 7.8)	0.040
Mean total LOS (days)	14.5 (SD 23.0)	16.2 (SD 28.1)	12.4 (SD 14.6)	0.050
RTS	6.15 (SD 3.1)	5.81 (SD 3.30)	6.55 (SD 2.82)	0.007
Mechanism of injury				
Blunt	467 (91.9%)	248 (90.5%)	219 (93.6%)	
Penetrating	29 (5.7%)	21 (7.7%)	8 (3.4%)	
Burns	12 (2.4%)	5 (1.8%)	7 (3.0%)	
Mortality	25 (4.9%)	15 (5.5%)	10 (4.3%)	0.682
Readmission*	19 (4.0%)	9 (3.5%)	10 (4.5%)	0.642

*ICU* Intensive Care Unit, *ISS* Injury Severity Score, *LOS* Length of Stay, *RTS* Revised Trauma Score, *TTL* Trauma team leader.

*Unplanned readmission within 60 days of discharge.

Approximately half of the cases (53.9%, n = 274) had a TTL present. The TTL and non-TTL groups were comparable in terms of sex, age, mechanism of injury and mortality (Table 1). The RTS was lower and ISS higher in the TTL group compared to the non-TTL group (5.81 vs. 6.55, p = 0.007 and 25.4 vs. 23.5, p = 0.045 respectively), indicating a more severely injured patient population in the TTL group.

### ATLS compliance rates

The compliance rates for ATLS protocols were based on the completion of 11 items for the primary survey and 4 items for the secondary survey that were chosen a priori by a group of trauma surgeons based on ATLS guidelines (Table 2). The median rates of completion for primary and secondary survey items for all patients were 81.8% (9 out of 11 items) and 75% (3 out of 4 items) respectively (Table 3). Compliance rate for completion of the primary survey was significantly higher (p = 0.003) for the TTL group (median of 10 out of 11 items, 90.9%) compared to the non-TTL group (median of 9 out of 11 items, 81.8%). Compliance rate for completion of the secondary survey was also significantly higher (p = 0.004) for the TTL group (median of 4 of 4 items, 100%) compared to the non-TTL group (median of 3 out of 4 items, 75%). Specifically, insertion of two large bore IVs 16 gauge or larger (TTL 68.2% vs. non-TTL 57.7%, p = 0.014), performance of the digital rectal exam (DRE) (TTL 64.6% vs. non-TTL 54.7%, p = 0.023), and performance of the head to toe exam (TTL 77.0% vs. 67.1%, p = 0.013) were higher in the TTL group (Tables 2 and 3).

**Table 2:** Compliance rates for primary survey, secondary survey and adjuncts

	**All patients (%)**	**TTL (%)**	**Non-TTL (%)**	**p-value**
*Primary survey*				
Airway patent	505 (99.4)	272 (99.3)	233 (99.6)	1.000
C spine immobilized	429 (84.4)	229 (83.6)	200 (85.5)	0.577
Chest auscultation	499 (98.2)	269 (98.2)	230 (98.3)	1.000
Chest palpation	295 (58.1)	163 (59.5)	132 (56.4)	0.483
Abdominal exam	499 (98.2)	270 (98.5)	229 (97.9)	0.739
Pelvis stability	333 (65.6)	183 (66.8)	150 (64.1)	0.525
Long bones exam	435 (85.6)	234 (85.4)	201 (85.9)	0.874
Two large bore IVs	322 (63.4)	187 (68.2)	135 (57.7)	0.014
Gross motor exam	439 (86.4)	243 (88.7)	196 (83.8)	0.106
Log roll	401 (78.9)	223 (81.4)	178 (76.1)	0.143
Digital rectal exam	305 (60.0)	177 (64.6)	128 (54.7)	0.023
*Secondary survey and adjuncts*				
Head to toe exam	368 (72.4)	211 (77.0)	157 (67.1)	0.013
AMPLE history	466 (91.7)	247 (90.1)	219 (93.6)	0.160
Trauma panel	440 (86.6)	240 ( 87.6)	200 (85.5)	0.484
Blood gas	357 (70.3)	197 (71.9)	160 (68.4)	0.387
*Diagnostic imaging*				
CXR	445 (87.6)	242 (88.3)	203 (86.8)	0.593
C spine XR	325 (64.0)	152 (55.5)	173 (73.9)	<0.001
Pelvis XR	281 (55.3)	136 (49.6)	145 (62.0)	0.005
CT head	375 (73.8)	197 (71.9)	178 (76.1)	0.287
CT chest	372 (73.2)	194 (70.8)	178 (76.1)	0.182
CT ab/pelvis	368 (72.4)	194 (70.8)	174 (74.4)	0.371
CT C spine	269 (53.0)	137 (50.0)	132 (56.4)	0.149

*Ab/Pelvis* Abdomen and pelvis, *AMPLE* Allergy, medication, past medical history, last meal, events, *C spine* Cervical spine, *CXR* Chest XR.

**Table 3:** Completion rates for primary and secondary surveys

	**Median (%)**	**p-value**
Primary survey*		
All patients	9 (81.8)	
TTL	10 (90.9)	0.003
Non-TTL	9 (81.8)	
*Secondary survey^*		
All patients	3 (75)	
TTL	4 (100)	0.004
Non-TTL	3 (75)	

*Out of a total of eleven items.

^Out of a total of four items.

### Time to diagnostic imaging

Mean times from arrival to the ED to performance of various diagnostic studies were obtained. Times to pelvis xray (68min vs. 107min, p = 0.007), CT of the chest (133min vs. 172min, p = 0.005), and CT of the abdomen and pelvis (136min vs. 173min, p = 0.013) were significantly faster for the TTL group compared to the non-TTL group (Table 4).

**Table 4:** Times to diagnostic imaging

**Diagnostic test**	**TTL involved**	**Non-TTL**	**p-value**
	**Mean time (min)**	**Mean time (min)**	
	**(SD) (min)**	**(SD) (min)**	
Chest X-ray	88 (172)	99 (157)	0.466
Pelvis X-ray	68 (77)	107 (160)	0.007
C spine X-ray	98 (134)	115 (146)	0.276
CT head	111 (109)	129 (82)	0.068
CT chest	133 (130)	172 (136)	0.005
CT ab/pelvis	136 (133)	173 (144)	0.013
CT C spine	131 (134)	166 (142)	0.054

*Ab/Pelvis* Abdomen and pelvis, *C spine* Cervical spine.

### Major outcome measures and readmission rate

Patients from the TTL group required significantly longer ICU LOS compared to the non-TTL group (mean 4.5 days vs. 2.9 days, p = 0.040). Although not statistically significant, the total LOS was also higher for the TTL group compared to the non-TTL group (16.2 days vs. 12.4 days, p = 0.050). There is no difference in mortality between the two groups (TTL 5.5% vs. non-TTL 4.3%, p = 0.682). The overall rate of unplanned readmission within 60 days was 4.0% (19 out of 477 patients), and the rates were not significantly different between the TTL group (3.5%, 9 out of 257 patients) and non-TTL group (4.5%, 10 out of 220 patients; p = 0.642) (Table 1).

## Discussion

ATLS provide a common framework and organized approach to trauma resuscitations, and has been shown to improve outcomes [4,5]. Studies have demonstrated the effectiveness of ATLS training on improving the quality of diagnostic and therapeutic procedures and decreasing mortality rate [4,5]. ATLS training and implementation, as a part of a well-organized trauma system, can improve outcomes of trauma patients [12-19].

As with any quality assessment, the results from this study demonstrated a need to improve overall ATLS compliance at our institution. However, the compliance rates for primary and secondary surveys at our institution were similar or slightly higher compared to other studies [9-11]. Santora *et al.*[9] found an overall deviation rate of 23% from ATLS protocols in their study using video assessment of trauma resuscitations, while the overall compliance rate for ATLS was only 53% in the study by Spanjersberg *et al.*[10]. In our study, the presence of a TTL during trauma resuscitation led to a significantly higher compliance rate for primary and secondary surveys, and also increased efficiency of resuscitation as demonstrated by the decrease in time to diagnostic imaging compared to the absence of a TTL. Time for CT acquisition for trauma patients range widely in the literature, from 17 to 197 minutes [20-24], and there is no definition for acceptable time to completion of diagnostic imaging in trauma patients. The mean times from patient arrival to completion of CT scans in our center were within the time frame reported by other studies; however, times to completion of xrays were often delayed. Although CT acquisition time has not been directly linked to affect major outcomes such as mortality or LOS, faster CT acquisition may be associated with time reduction to live-saving interventions [25].

There were certain areas in the primary and secondary surveys where the non-TTL group seemingly out-performed the TTL group, such as the utilization of basic radiography. Although plain C spine and pelvic xrays are part of the ATLS algorithm, with the availability of CT scanners, they have a diminishing role for hemodynamically stable blunt trauma patients with a severe mechanism of injury [26-28]. Several studies have found that pelvic xray has low sensitivity compared to CT of the pelvis, and may be omitted in hemodynamically stable blunt trauma patients who will have CT of the abdomen and pelvis [26-28]. Similarly, CT C spine is superior to C spine xray (due to frequent inadequate views) [29-31], and is replacing C spine xrays in many trauma centers [32,33]. On the basis of the current evidence, a TTL may have chosen to omit C spine and pelvic xrays on patients who were receiving CT C spine, abdomen and pelvis. This may have potentially reduced redundant imaging and unnecessary delays in the trauma resuscitation area. Overall, the times to imaging, however, were longer than expected, and could be improved upon as a quality initiative.

Our study showed a significantly longer ICU stay and a trend for longer hospital stay for the TTL group compared to the non-TTL group. This may be accounted for by the lower RTS and higher ISS in the TTL group compared the non-TTL group, indicating a higher severity of injuries in the TTL group. Although we have not been able to demonstrate a direct link between ATLS compliance and mortality, the efficiency of trauma resuscitations was improved by the presence of a TTL as demonstrated by the decreased time from patient arrival to performance of various diagnostic imaging.

Studies on medical and surgical patients have shown that the rate of early readmission is associated with quality of inpatient care [34]. In addition, the American College of Surgeons’ Committee on Trauma has recommended that readmissions due to complications should be an audit filter in the quality of care monitors [35]. We have therefore used readmission rate as a surrogate marker for quality of care delivered to trauma patients. Previous studies on early readmission for trauma patients showed a readmission rate ranging from 1.2 to 10.9% [36-38], which is comparable to this study. Several factors are associated with readmissions after trauma, in particular, severity of injuries [36,38]. One would expect the TTL group to have a higher readmission rate compared to the non-TTL group due to a higher severity of injuries. The fact that the readmission rates were similar between the two groups may indicate a positive effect on patient care with the presence of a TTL, since other aspects of inpatient care were standardized for both groups of patients. Further studies are required to determine the exact impact of TTL on process of care and readmission rates.

Given the findings of this study and evidence in the literature, the consistent presence of a TTL during resuscitations of major trauma patients is important for maintaining compliance with ATLS protocols. Although one can postulate that better compliance rates for performing the primary and secondary surveys in the TTL group compared to the non-TTL group were based on increased leadership abilities, it is possible that the non-TTL group had less resources and manpower available leading to lower compliance.

At the time of the study, TTLs were composed of a multidisciplinary group of ED physicians, general surgeons, and one neurosurgeon. All of the TTLs have ATLS certification, and are involved in ATLS education, quality assurance, and research. As a whole, this group is more likely to be familiar with up to date ATLS protocols and evidence-based trauma studies, and see a higher volume of major trauma patients. The TTL serves an important role in trauma resuscitations by promoting leadership, team cohesiveness, and communication within the multidisciplinary team, to ensure efficiency and efficacy of the resuscitation [19]. TTLs can also reinforce protocol-driven approaches to trauma care that improve patient care [39]. Gerardo *et al.*[19] demonstrated a reduction in mortality rate, most notably in the most severely injured patients, when a dedicated trauma team was implemented in a Level I trauma center.

During the time period examined in our institution, a TTL was present in only half of the trauma resuscitations. Reports from UK and Australia found similar rates of involvement by the trauma team and TTL [40,41]. We believe there are two contributing factors: gaps in the TTL call scheduling, and lack of TTL notification as a part of activation of the trauma team. Reviewing the TTL call schedule at the study period, an average of 31% of shifts were not covered by a TTL (data not shown). At times when a TTL was not scheduled, the leadership role fell onto the attending ED physician, the attending surgeon, or senior general surgery resident. At our institution, TTL coverage can be improved by recruitment and retention of qualified physicians interested in trauma, and by including non-surgeons such as anesthetists, emergency physicians and intensivists. Although this study was not designed to measure the appropriateness of TTL or trauma team activation, there appears to be an element of under triage regarding trauma team activation and involvement of the TTL on call. Some of the current barriers include the lack of understanding surrounding the role of a TTL, interruptions in trauma resuscitations especially when a TTL arrives late, as well as the impression of chaos and “too many people” when the trauma team is activated. Various studies have demonstrated that appropriate activation of the trauma team can improve outcomes [42,43], and under-triaged trauma patients are associated with a high risk of mortality [42]. In order to promote a culture of safety, there needs to be ongoing education on TTL activation criteria for all staff involved in trauma resuscitations. Secondly, education should also focus on the benefits of TTL activation versus harm of “under-call”. Lastly, ongoing audits should target TTL activation rate and timely feedback should be provided to all players in trauma resuscitations to ensure proper and consistent TTL activation.

Attrition of ATLS knowledge may also have contributed to poor compliance. In a study by *Ali et al*. [6], significant attrition rates of cognitive knowledge and skills was evident as early as 6 months after participants completed an ATLS course. The same group showed the attrition rate was higher for participants from low-volume centers compared to high-volume centers [7]. To address this issue, continued trauma education for all members of the trauma team should be actively encouraged and supported. This can take the form of multidisciplinary trauma simulations, maintenance of ATLS certification, other advanced courses in trauma, and attendance at trauma conferences. Additional training in trauma team crisis resource management may improve team cohesiveness, and the requirement of all physicians involved in trauma resuscitations to maintain active ATLS certification should also be established.

This study has a number of limitations. Trauma resuscitations are highly dynamic and as such not all actions performed were adequately documented with certainty. The chart review revealed a lack of time entries in many areas and this has made time-dependent outcome measures hard to gather. In particular, the rate of completion of FAST exams and time to FAST exam could not be reliably obtained from the chart review due to inconsistent record keeping. The study only reviewed data from a one-year period and as a result may not have the necessary power to show differences in major outcomes between the TTL compared to the non-TTL groups. However, we have obtained important data on the performance outcomes in the form of ATLS compliance rate, readmission rate, and indirect measure of efficiency of trauma resuscitations via times to diagnostic imaging. Additionally, we have also identified areas of future improvement with this quality assessment, and hope that other institutions will use our study as a model to promote their own quality reviews.

## Conclusions

We have demonstrated that TTL involvement significantly improved compliance with many aspects of ATLS, and increased the efficiency of trauma resuscitations by decreasing mean time to diagnostic imaging. There is an acute need to improve compliance with ATLS protocols at our center as well as increase TTL involvement in major traumas at our institution. The reluctance in the hospital culture to activate the trauma team and TTL should be targeted with education around the importance of trauma team activation and involvement of TTL, as well as promotion of a culture of safety. Deficiencies found in this study will guide future quality control initiatives for trauma management.

## Abbreviations

ATLS: Advanced trauma life support; ATR: Alberta trauma registry; DRE: Digital rectal exam; ED: Emergency department; ICU: Intensive care unit; ISS: Injury severity score; LOS: Length of stay; RTS: Revised trauma score; TTL: Trauma team leader.

## Competing interests

The authors declare that they have no competing interests.

## Authors’ contributions

BT and SW conceived and designed the study, and drafted the manuscript. BT was responsible for data collection. JM was responsible for statistical analysis. PE, JM and DPG helped with the drafting and editing of the manuscript. All authors read and approved the final manuscript.

## References

[B1] SMARTRISK The Economic Burden of Injury in Canada 2009 Toronto, ON: SMARTRISK

[B2] McDermott FT Cordner SM Tremayne AB A “before and after” assessment of the influence of the new Victorian trauma care system (1997–1998 vs 2001–2003) on the emergency and clinical management of road traffic fatalities in Victoria. Report of the Consulatative Committee on Road Traffic Fatalities 2003 Victorian Institute for Forensic Medicine: Melbourne, Australia

[B3] American College of Surgeons Advanced Trauma Life Support Program for Doctors: 9th ed 2012 Chicago: American College of Surgeons

[B4] van Olden GD Meeuwis JD Bolhuis HW Boxma H Goris RJ Advanced trauma life support study: quality of diagnostic and therapeutic procedures J Trauma 2004 8 381 384 10.1097/01.TA.0000096645.13484.E6 15345989

[B5] van Olden GD Meeuwis JD Bolhuis HW Boxma H Goris RJ Clinical impact of advanced trauma life support Am J Emerg Med 2004 8 522 525 10.1016/j.ajem.2004.08.013 15666253

[B6] Ali J Cohen R Adam R Gana TJ Pierre I Ali E Bedaysie H West U Winn J Attrition of cognitive and trauma management skills after the Advanced Trauma Life Support (ATLS) course J Trauma 1996 8 860 866 10.1097/00005373-199606000-00002 8656470

[B7] Ali J Howard M Williams J Is attrition of advanced trauma life support acquired skills affected by trauma patient volume? Am J Surg 2002 8 142 145 10.1016/S0002-9610(01)00862-5 11918877

[B8] McCrum ML McKee J Lai M Staples J Switzer N Widder SL ATLS adherence in the transfer of rural trauma patients to a level I facility Injury 2012 in press 10.1016/j.injury.2012.05.009 22658421

[B9] Santora TA Trooskin SZ Blank CA Clarke JR Schinco MA Video assessment of trauma response: adherence to ATLS protocols Am J Emerg Med 1996 8 564 569 10.1016/S0735-6757(96)90100-X 8857806

[B10] Spanjersberg WR Bergs EA Mushkudiani N Klimek M Schipper IB Protocol compliance and time management in blunt trauma resuscitation Emerg Med J 2009 8 23 27 19104091 10.1136/emj.2008.058073

[B11] Fitzgerald M Gocentas R Dziukas L Cameron P Mackenzie C Farrow N Using video audit to improve trauma resuscitation–time for a new approach Can J Surg 2006 8 208 211 16749983 PMC3207597

[B12] Shackford SR Hollingworth-Fridlund P Cooper GF Eastman AB The effect of regionalization upon the quality of trauma care as assessed by concurrent audit before and after institution of a trauma system: a preliminary report J Trauma 1986 8 812 820 10.1097/00005373-198609000-00006 3746956

[B13] McDermott F Cordner S Winship V Addressing inadequacies in Victoria’s trauma system: responses of the Consultative Committee on Road Traffic Fatalities and Victorian trauma services Emerg Med Australas 2010 8 224 231 10.1111/j.1742-6723.2010.01288.x 20497210

[B14] Simons R Eliopoulos V Laflamme D Brown DR Impact on process of trauma care delivery 1 year after the introduction of a trauma program in a provincial trauma center J Trauma 1999 8 811 815 10.1097/00005373-199905000-00008 10338397

[B15] Alberts KA Bellander BM Modin G Improved trauma care after reorganisation: a retrospective analysis Eur J Surg 1999 8 426 430 10.1080/110241599750006640 10391157

[B16] Barquist E Pizzutiello M Tian L Cox C Bessey PQ Effect of trauma system maturation on mortality rates in patients with blunt injuries in the Finger Lakes Region of New York State J Trauma 2000 8 63 69 10.1097/00005373-200007000-00009 10912859

[B17] Nathens AB Jurkovich GJ Rivara FP Maier RV Effectiveness of state trauma systems in reducing injury-related mortality: a national evaluation J Trauma 2000 8 25 30 10.1097/00005373-200001000-00005 10647561

[B18] Abernathy JH 3rd McGwin G Jr Acker JE 3rd Rue LW 3rd Impact of a voluntary trauma system on mortality, length of stay, and cost at a level I trauma center Am Surg 2002 8 182 192 11842968

[B19] Gerardo CJ Glickman SW Vaslef SN Chandra A Pietrobon R Cairns CB The rapid impact on mortality rates of a dedicated care team including trauma and emergency physicians at an academic medical center J Emerg Med 2011 8 586 591 10.1016/j.jemermed.2009.08.056 20022198

[B20] Easton R Sisak K Balogh ZJ Time to computed tomography scanning for major trauma patients: the Australian reality ANZ J Surg 2012 8 644 647 10.1111/j.1445-2197.2012.06150.x 22882660

[B21] Lee KL Graham CA Lam JM Yeung JH Ahuja AT Rainer TH Impact on trauma patient management of installing a computed tomography scanner in the emergency department Injury 2009 8 873 875 10.1016/j.injury.2008.12.001 19394016

[B22] Wurmb TE Fruhwald P Hopfner W Keil T Kredel M Brederlau J Roewer N Kuhnigk H Whole-body multislice computed tomography as the first line diagnostic tool in patients with multiple injuries: the focus on time J Trauma 2009 8 658 665 10.1097/TA.0b013e31817de3f4 19276734

[B23] Fung Kon Jin PH Goslings JC Ponsen KJ van Kuijk C Hoogerwerf N Luitse JS Assessment of a new trauma workflow concept implementing a sliding CT scanner in the trauma room: the effect on workup times J Trauma 2008 8 1320 1326 10.1097/TA.0b013e318059b9ae 18469657

[B24] Fung Kon Jin PH van Geene AR Linnau KF Jurkovich GJ Ponsen KJ Goslings JC Time factors associated with CT scan usage in trauma patients Eur J Radiol 2009 8 134 138 10.1016/j.ejrad.2008.06.022 18657921

[B25] Bernhard M Becker TK Nowe T Mohorovicic M Sikinger M Brenner T Richter GM Radeleff B Meeder PJ Buchler MW Bottiger BW Martin E Gries A Introduction of a treatment algorithm can improve the early management of emergency patients in the resuscitation room Resuscitation 2007 8 362 373 10.1016/j.resuscitation.2006.09.014 17287064

[B26] Guillamondegui OD Pryor JP Gracias VH Gupta R Reilly PM Schwab CW Pelvic radiography in blunt trauma resuscitation: a diminishing role J Trauma 2002 8 1043 1047 10.1097/00005373-200212000-00002 12478025

[B27] Hilty MP Behrendt I Benneker LM Martinolli L Stoupis C Buggy DJ Zimmermann H Exadaktylos AK Pelvic radiography in ATLS algorithms: A diminishing role? World J Emerg Surg 2008 8 11 10.1186/1749-7922-3-11 18318904 PMC2311282

[B28] Kessel B Sevi R Jeroukhimov I Kalganov A Khashan T Ashkenazi I Bartal G Halevi A Alfici R Is routine portable pelvic X-ray in stable multiple trauma patients always justified in a high technology era? Injury 2007 8 559 563 10.1016/j.injury.2006.12.020 17303137

[B29] Bailitz J Starr F Beecroft M Bankoff J Roberts R Bokhari F Joseph K Wiley D Dennis A Gilkey S Erickson P Raksin P Nagy K CT should replace three-view radiographs as the initial screening test in patients at high, moderate, and low risk for blunt cervical spine injury: a prospective comparison J Trauma 2009 8 1605 1609 10.1097/TA.0b013e3181a5b0cc 19509621

[B30] Holmes JF Akkinepalli R Computed tomography versus plain radiography to screen for cervical spine injury: a meta-analysis J Trauma 2005 8 902 905 10.1097/01.TA.0000162138.36519.2A 15920400

[B31] Duane TM Dechert T Brown H Wolfe LG Malhotra AK Aboutanos MB Ivatury RR Is the lateral cervical spine plain film obsolete? J Surg Res 2008 8 267 269 10.1016/j.jss.2008.02.062 18498879

[B32] Widder S Doig C Burrowes P Larsen G Hurlbert RJ Kortbeek JB Prospective evaluation of computed tomographic scanning for the spinal clearance of obtunded trauma patients: preliminary results J Trauma 2004 8 1179 1184 10.1097/01.TA.0000130758.71098.78 15211122

[B33] Hennessy D Widder S Zygun D Hurlbert RJ Burrowes P Kortbeek JB Cervical spine clearance in obtunded blunt trauma patients: a prospective study J Trauma 2010 8 576 582 10.1097/TA.0b013e3181cf7e55 20220418

[B34] Ashton CM Del Junco DJ Souchek J Wray NP Mansyur CL The association between the quality of inpatient care and early readmission: a meta-analysis of the evidence Med Care 1997 8 1044 1059 10.1097/00005650-199710000-00006 9338530

[B35] American College of Surgeons Committee on Trauma: Resources for optimal care of the injured patient 1993 Chicago: American College of Surgeons 10134114

[B36] Battistella FD Torabian SZ Siadatan KM Hospital readmission after trauma: an analysis of outpatient complications J Trauma 1997 8 1012 1016 10.1097/00005373-199706000-00004 9210533

[B37] Moore L Thomas Stelfox H Turgeon AF Nathens AB Sage NL Emond M Bourgeois G Lapointe J Gagne M Rates, patterns, and determinants of unplanned readmission after traumatic injury: a multicentre cohort study Ann Surg 2013 in press 10.1097/SLA.0b013e31828b0fae 23478531

[B38] Morris DS Rohrbach J Sundaram LM Sonnad S Sarani B Pascual J Reilly P Schwab CW Sims C Early hospital readmission in the trauma population: Are the risk factors different? Injury 2013 in press 10.1016/j.injury.2013.04.029 PMC4149179 23726120

[B39] Driscoll PA Vincent CA Variation in trauma resuscitation and its effect on patient outcome Injury 1992 8 111 115 10.1016/0020-1383(92)90044-S 1572705

[B40] Findlay G Martin IC Carter S Smith N Weyman D Mason M Trauma: Who cares? A report of the National Confidential Enquiry into Patient Outcome and Death (2007) 2007 London, UK: NCEPOD

[B41] Wong K Petchell J Trauma teams in Australia: a national survey ANZ J Surg 2003 8 819 825 10.1046/j.1445-2197.2003.02782.x 14525574

[B42] Rainer TH Cheung NK Yeung JH Graham CA Do trauma teams make a difference? A single centre registry study Resuscitation 2007 8 374 381 10.1016/j.resuscitation.2006.10.011 17289243

[B43] Petrie D Lane P Stewart TC An evaluation of patient outcomes comparing trauma team activated versus trauma team not activated using TRISS analysis. Trauma and Injury Severity Score J Trauma 1996 8 870 873 discussion 873–5 10.1097/00005373-199611000-00020 8913219

